# Pathways from research to sustainable development: Insights from ten research projects in sustainability and resilience

**DOI:** 10.1007/s13280-023-01968-4

**Published:** 2024-02-07

**Authors:** Anna Scaini, Joseph Mulligan, Håkan Berg, Albert Brangarí, Vera Bukachi, Sebastian Carenzo, Da Chau Thi, Colin Courtney-Mustaphi, Anneli Ekblom, Hanne Fjelde, Mathias Fridahl, Anders Hansson, Lettice Hicks, Mattias Höjer, Benard Juma, Jaan-Henrik Kain, Rebecca W. Kariuki, Soben Kim, Paul Lane, Ainara Leizeaga, Regina Lindborg, John Livsey, Steve W. Lyon, Rob Marchant, Jennifer R. McConville, Linus Munishi, David Nilsson, Luke Olang, Stefan Olin, Lennart Olsson, Peter Msumali Rogers, Johannes Rousk, Hans Sandén, Nophea Sasaki, Anna Shoemaker, Benjamin Smith, Lan Thai Huynh Phuong, Ana Varela Varela, Manjunatha Venkatappa, Giulia Vico, Nina Von Uexkull, Christine Wamsler, Menale Wondie, Patrick Zapata, María José Zapata Campos, Stefano Manzoni, Anna Tompsett

**Affiliations:** 1 Department of Physical Geography, Stockholm University, 10691 Stockholm, Sweden; 2 Bolin Centre for Climate Research, Stockholm University, 10691 Stockholm, Sweden; 3 Kounkuey Design Initiative (KDI), Los Angeles, CA USA; 4 Department of Sustainable Development, Environmental Science and Engineering (SEED), KTH Royal Institute of Technology, Stockholm, Sweden; 5 Microbial Ecology, Department of Biology, Lund University, Lund, Sweden; 6 University College London, London, UK; 7 Instituto de Estudios sobre la Ciencia y la Tecnología, Universidad Nacional de Quilmes/CONICET, Buenos Aires, Argentina; 8 Faculty of Applied Sciences, Ton Duc Thang University, Ho Chi Minh City, Vietnam; 9 Geoecology, Department of Environmental Sciences, University of Basel, Klingelbergstrasse 27, 4056 Basel, Switzerland; 10 Center for Water Infrastructure and Sustainable Energy (WISE) Futures, Nelson Mandela African Institution of Science and Technology, P.O. Box 9124, Nelson Mandela, Tengeru Tanzania; 11 Department of Archaeology and Ancient History, Uppsala University, 752 38 Uppsala, Sweden; 12 Department of Peace and Conflict Research, Uppsala University, Uppsala, Sweden; 13 Unit of Environmental Change, Department of Thematic Studies, Institution of Arts and Sciences, Linköping University, 581 83 Linköping, Sweden; 14 Division of Strategic Sustainability Studies, Environmental Science and Engineering (SEED), KTH Royal Institute of Technology, Stockholm, Sweden; 15 Department of Civil and Construction Engineering, Technical University of Kenya, P.O Box 52428-00200, Nairobi, Kenya; 16 Gothenburg Research Institute, University of Gothenburg, 405 30 Göteborg, Sweden; 17 School of School of Sustainability, Arizona State University, Arizona, USA; 18 School of Life Sciences and Bio-Engineering, Nelson Mandela African Institution of Science and Technology, P.O Box 447, Arusha, Tanzania; 19 Faculty of Forestry Science) Dangkor, Royal University of Agriculture, P.O. Box 2696, Phnom Phnom, Cambodia; 20 Department of Archaeology, University of Cambridge, Cambridge, UK; 21 Department of Earth and Environmental Sciences, The University of Manchester, Michael Smith Building, Manchester, UK; 22 School of Environment and Natural Resources, Ohio State University, Columbus, OH 43210 USA; 23 Department of Energy and Technology, Swedish University of Agricultural Sciences (SLU), 75007 Uppsala, Sweden; 24 Division of History of Science, Technology and Environment, KTH Royal Institute of Technology, Stockholm, Sweden; 25 Department of Biosystems and Environmental Engineering, Technical University of Kenya, P.O. Box 52428-00200, Nairobi, Kenya; 26 Department of Physical Geography and Ecosystem Science, Lund University, 22362 Lund, Sweden; 27 Lund University Centre for Sustainability Studies (LUCSUS), Lund University, Box 170, 22100 Lund, Sweden; 28 Institute of Resource Assessment, University of Dar es Salaam, Dar es Salaam, Tanzania; 29 University of Natural Resources and Life Sciences (BOKU), Vienna, Austria; 30 Natural Resources Management, Asian Institute of Technology, P.O. Box 4, Klong Luang, 12120 Pathum Thani Thailand; 31 Hawkesbury Institute for the Environment, Western Sydney University, Richmond, NSW Australia; 32 Department of Rural Development and Natural Resources Management, An Giang University, Long Xuyên, 90000 An Giang Province Vietnam; 33 Vietnam National University, Ho Chi Minh City, 70000 Vietnam; 34 London School of Economics, Department of Geography and Environment, London, UK; 35 LEET Intelligence Co., Ltd., Suan Prikthai, Muang Pathum Thani, 12000 Pathum Thani Thailand; 36 Department of Crop Production Ecology, Swedish University of Agricultural Sciences (SLU), 750 07 Uppsala, Sweden; 37 Centre of Natural Hazards and Disaster Science (CNDS), Uppsala, Sweden; 38 Amhara Regional Agricultural Research Institute (ARARI), Bahir Dar, Ethiopia; 39 School of Public Administration, University of Gothenburg, Gothenburg, Sweden; 40 Department of Business Administration, School of Business, Economics and Law, University of Gothenburg, 40530 Gothenburg, Sweden; 41 Institute for International Economic Studies, Stockholm University, 10691 Stockholm, Sweden

**Keywords:** Climate change adaptation, Knowledge co-creation, Knowledge transfer, Resilience, Sustainable development goals, Upscaling

## Abstract

Drawing on collective experience from ten collaborative research projects focused on the Global South, we identify three major challenges that impede the translation of research on sustainability and resilience into better-informed choices by individuals and policy-makers that in turn can support transformation to a sustainable future. The three challenges comprise: (i) converting knowledge produced during research projects into successful knowledge application; (ii) scaling up knowledge in time when research projects are short-term and potential impacts are long-term; and (iii) scaling up knowledge across space, from local research sites to larger-scale or even global impact. Some potential pathways for funding agencies to overcome these challenges include providing targeted prolonged funding for dissemination and outreach, and facilitating collaboration and coordination across different sites, research teams, and partner organizations. By systematically documenting these challenges, we hope to pave the way for further innovations in the research cycle.

**Supplementary Information:**

The online version contains supplementary material available at 10.1007/s13280-023-01968-4.

## Introduction

Research funders are increasingly allocating funds toward sustainability and resilience research. The implicit or explicit goal of these funding initiatives is to produce knowledge that can help individuals and policy-makers to make better choices, in turn moving us closer to achieving our collective sustainability goals, as set out in international agreements (e.g., UNECE 1998; UN 2015, 2017). But there is much we do not know about translating research on sustainability and resilience into real-world impacts. What challenges are encountered? How prevalent are these challenges, and how do they vary across disciplines? How effective are the strategies that are commonly proposed to overcome these challenges? What more can funders and researchers do?

In this perspective paper, we draw on practical experience from ten research projects in sustainability and resilience—which together comprise the entire funded portfolio of a single call for proposals—to document and taxonomize the challenges encountered in translating sustainability and resilience research into real-world impact. The call for proposals was jointly funded by the Swedish International Development Agency (Sida) and by two Swedish Research Councils (Vetenskapsrådet and Formas) (Swedish Research Council Vetenskapsrådet Formas and Sida 2016). The agencies awarded approximately 6.2 million USD (54 million SEK) over up to 3 years across the ten funded projects. The authors of this perspective paper comprise members from all ten project teams, including all principal investigators.

Sustainability and resilience are terms with many definitions (UNWCED 1987; UNISDR 2009; Folke 2016; Clark and Harley 2020). The call for proposals, while not explicitly defining these terms, required projects to relate both to climate and environmental change and to poverty alleviation. This emphasis on poverty alleviation aligns with the most commonly used definition for sustainable development, the “Brundtland” definition (UNWCED 1987), which proposes that sustainable development should meet “the needs of the present without compromising the ability of future generations to meet their own needs” and given an implicit hierarchy of needs prioritizes “the essential needs of the poor.”

The portfolio of funded projects thus reflects the working definition of sustainability and resilience that Swedish policymakers and research funders had in mind when they designed the call for proposals and selected projects, as well as the ways in which researchers interpreted the call for proposals when they formulated their research projects. All projects self-identified as research in sustainability and resilience when they applied for the call.

**Fig. 1:**
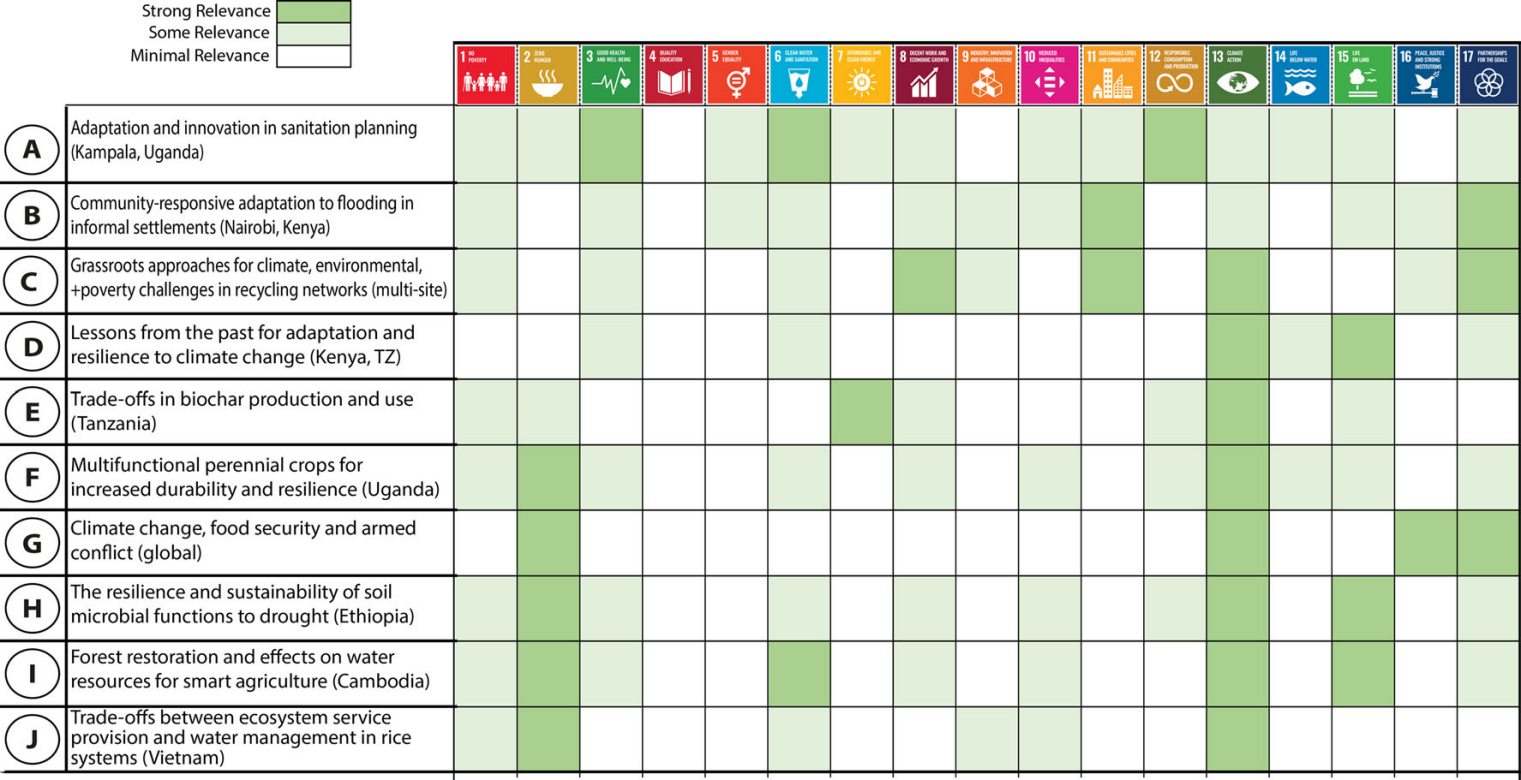
List of projects and relevance to SDGs as evaluated by the research teams at the end of the funding cycle

The ten research projects span a wide range of disciplines (Table 1) and scopes, as illustrated by the range of the United Nations Agenda 2030 Sustainable Development Goals (SDGs, UN 2015) to which the projects are relevant (Fig. 1). While the most relevant SDGs—climate action and zero hunger—reflect the core criteria of the call for funding, sixteen out of seventeen SDGs are relevant to at least one project. In our view, this diversity is intrinsic to the field of sustainability and resilience and helps illustrate how much potential scope there is for expanding knowledge in ways that will help us achieve social goals including the SDGs.

**Table 1:** Key information about the projects including research sites, disciplinary scope, and the identities of potential knowledge users

Project	Short title	Research sites	Researcher disciplines	Knowledge users	Key References
A	Adaptation and innovation in sanitation planning	Kampala, Uganda	Civil & Environmental Engineering, Biology, Architecture	Water utility providers, the Minister for Water, National agricultural research center and NGOs	Billger et al. (2020), McConville et al. (2020), Kain et al. (2021), McConville et al. (2022)
B	Community-responsive adaptation to flooding in informal settlements	Nairobi, Kenya	Economics, Civil Engineering, Hydrology, Urban Planning	Residents of informal settlements, practitioners (architects, engineers, landscape architects, planners), local government	Mulligan et al. (2016, 2019), Juma et al. (2021), Wamsler et al. (2022)
C	Grassroots approaches for climate, environmental, and poverty challenges in recycling networks	Multi-site	Anthropology, sociology, water and sanitation engineering, urban planning, human geography, public administration, business administration, political science	Waste pickers, waste picker organizations, residents of informal settlements, resident associations, municipal officers, politicians working, NGOs	Zapata Campos et al. (2020), Zapata Campos et al. (2023c), Zapata Campos et al. (2023b), Zapata Campos et al. (2023a)
D	Lessons from the past for adaptation and resilience to climate change	Kenya, Tanzania	Archaeology, Palaeoecology, Ecology, History, Geography, Environmental Sciences	Pastoralists, farmers, hunter-gatherers, wildlife managers, CBOs, NGOs, local government officers, tourism operators, heritage professionals, faith-based leaders, land use planners, socio-economic researchers, environmentalists	Courtney Mustaphi et al. (2019), Kariuki et al. (2021)
E	Trade-offs in biochar production and use	Tanzania	Social science (sociology, technology and social change, environmental change), agriculture science, soil science, botany	Farmers, local administrators, local NGOs	Fridahl et al. (2021), Hansson et al. (2021), Rogers et al. (2022)
F	Multifunctional perennial crops for increased durability and resilience	Uganda	Human ecology, human geography, sustainability science, physical geography	Farmers and Rural Advisory Services	Crews et al. (2018), Isgren et al. (2020)
G	Climate change, food security and armed conflict	Global	Peace and conflict research, sociology, anthropology	International organisations (United Nations Food and Agriculture Organisations (FAO)), governments	Von Uexkull and Buhaug (2021)
H	The resilience and sustainability of soil microbial functions to drought	Ethiopia	Ecology, Geosciences, Forestry, environmental science, agriculture	Practitioners (farmers, forestry practitioners). Local government. Regional farmers and forestry practitioners	Leizeaga et al. (2022)
I	Forest restoration and effects on water resources for smart agriculture	Cambodia	Ecology, Geosciences, Forestry	National government agencies, local government and community organisations, NGOs	Johansson et al. (2020), Venkatappa et al. (2020)
J	Trade-offs between ecosystem service provision and water management in rice systems	Vietnam	Engineering, hydrology, agriculture, environmental science	Farmers, local administrators	Livsey et al. (2021)

**Fig. 2:**
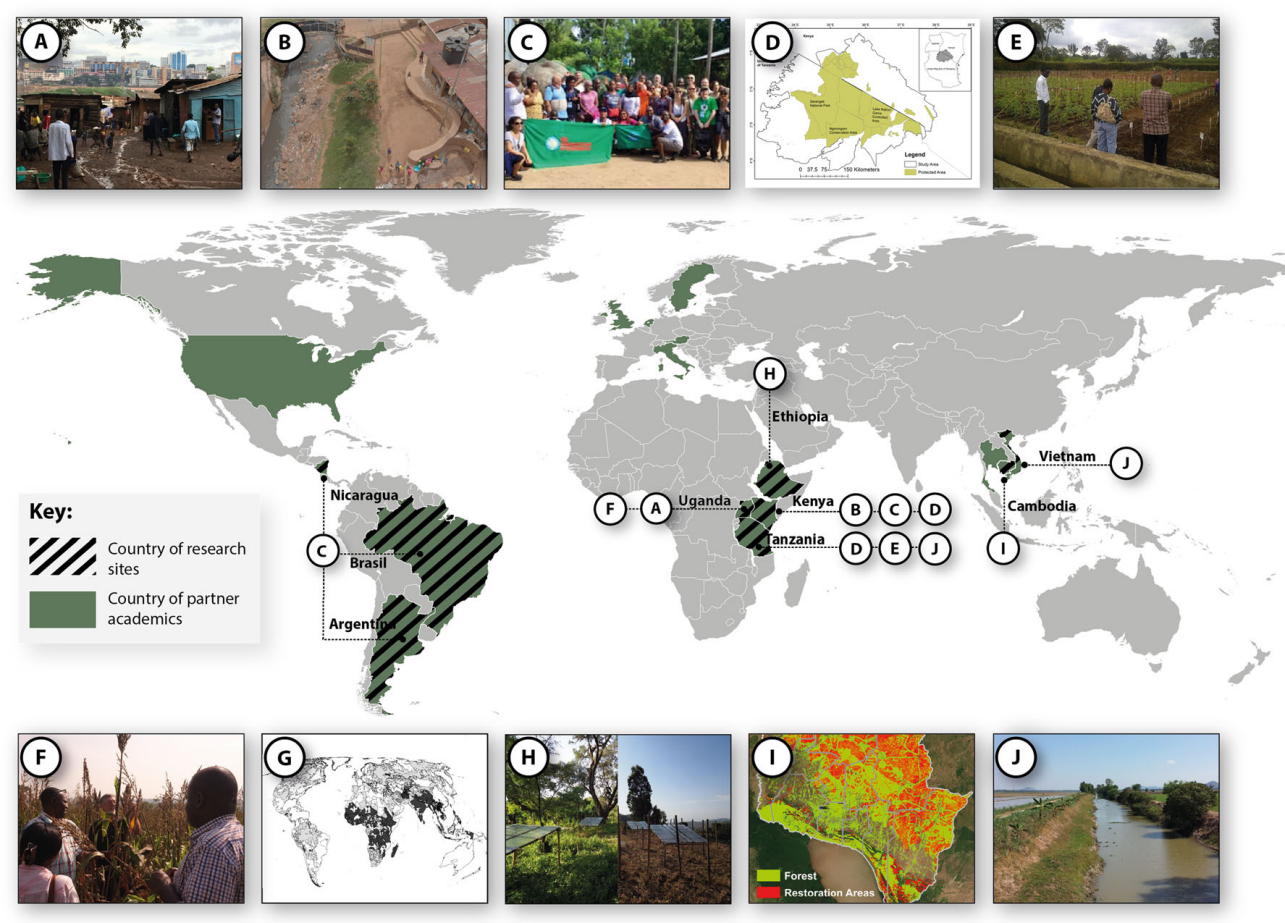
Research sites and countries where partner academics research. Images selected to visualize geographical scale of research projects. Photo credits: the authors. The authors give permission to use the photos

The call for proposals also required projects to be relevant to at least one low-income country, as defined by the Organization for Economic Co-operation and Development (OECD) Development Assistance Committee (OECD 2014). The projects studied the interactions between environmental and human systems at a variety of spatial scales. Nine out of ten projects focused on one or more specific geographical sites, many of which were in low-income countries, and one project was global in scope (Fig. 2). Each project entailed collaboration between researchers based at Swedish research institutions and researchers based in low or lower middle-income countries.

**Fig. 3:**
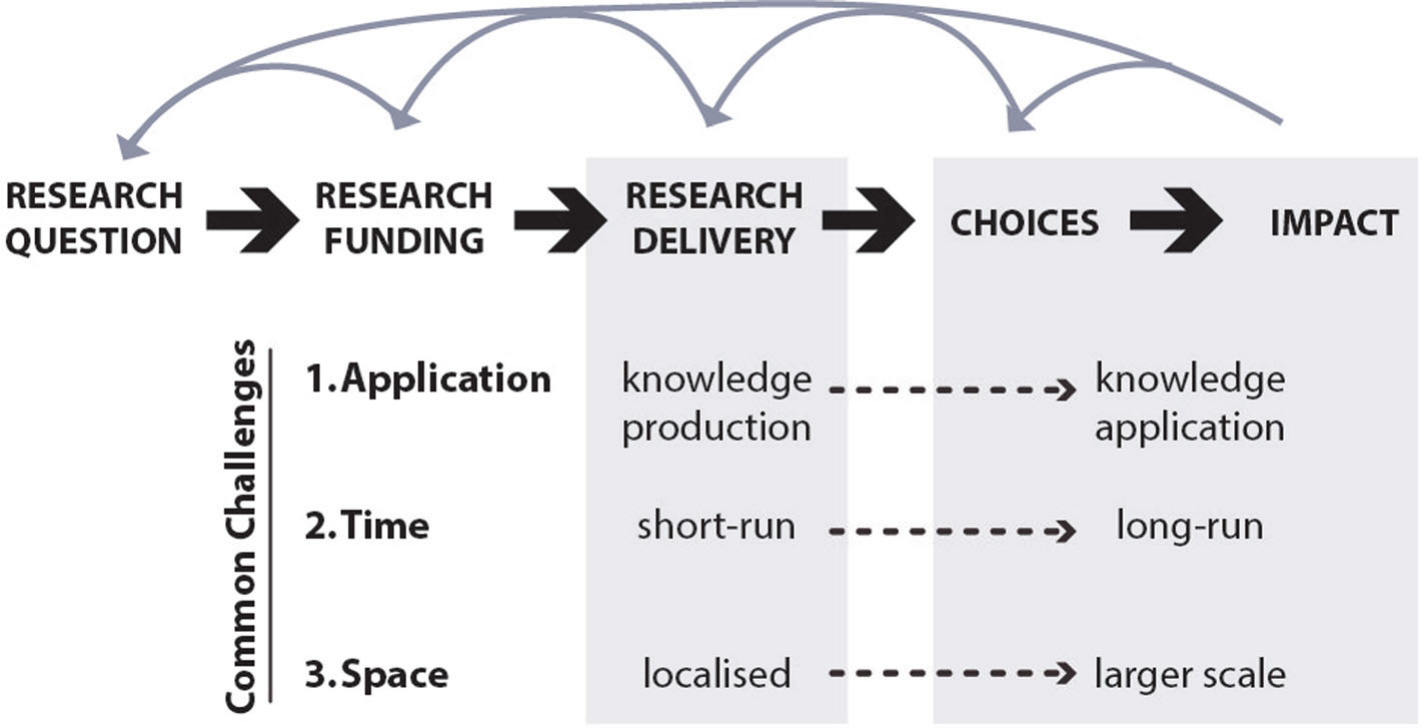
Schematic illustrating the three common challenges projects encountered in the transition from research to real-world impact (indicated by black dashed arrows). Gray arrows represent information flow feeding back to earlier stages in the transition

All ten project research teams came together for workshops at both the start and end of the funding cycle. At the end of the funding cycle, research teams presented progress to date and participated in reflective discussion sessions about how research can translate into real-world impact. Three major common challenges emerged from these discussions (Fig. 3). Each project later produced a brief case study highlighting the most salient challenges to their project. We refer to these case studies throughout the paper and provide them for reference in Supplementary Information. Strikingly, given the diversity highlighted above, most projects faced all three challenges, albeit to various extents.

The first challenge is the perennial problem of the *transition* from knowledge production into knowledge application (“Challenge I: Knowledge production to knowledge application” section). While the importance of this transition is far from unique to sustainability and resilience research, it is especially relevant to these fields, defined as they are “by the practical problems” they address (Clark and Harley 2020). In particular, the evaluation criteria for the projects in this study required projects to demonstrate their relevance to poverty reduction and sustainable development goals in low-income countries.

A widely held view is that a successful transition from knowledge production to application requires engagement with potential knowledge users at all stages of the research process, to ensure that research addresses questions that are valuable for user communities and to increase the likelihood that users can and will make use of the knowledge produced (Lemos et al. 2012; Bansard et al. 2019). Indeed, many posit that knowledge should be co-created or co-produced in collaboration with knowledge users, ideally reducing or eliminating entirely the issue of knowledge transition (White et al. 2001; Cash et al. 2006; Wyborn et al. 2019; Miller and Wyborn 2020), although others note that co-production may not be applicable or desirable in all contexts (Contandriopoulos et al. 2010; Lemos et al. 2018; Oliver et al. 2019; Wamsler et al. 2020). The potential knowledge users in the projects we study span the gamut from residents of informal settlements in Kenya to rural farmers in Ethiopia and from local government administrators in Uganda to the policy-makers that shape global treaties on climate change. This diversity notwithstanding, all projects prioritized collaboration and engagement with knowledge users, alongside North–South academic collaboration. While not all projects could aspire to co-production of knowledge with all potential users, many built on longstanding collaborations with knowledge user organizations, and many also specifically developed innovative approaches to user engagement. These features place the projects relatively high in an implicit hierarchy of strategies for user engagement designed to improve links between knowledge and action in sustainability research (Van Kerkhoff and Lebel 2006). Despite this commitment to engagement with knowledge users, most projects encountered persistent unresolved barriers to knowledge application, suggesting that as others have previously posited, researcher engagement with knowledge users may form only part of the solution to creating useable knowledge (Höchtl et al. 2006; Armitage et al. 2011; Lövbrand 2011; Oliver et al. 2019; Wyborn et al. 2019).

The second challenge is about *time* (“Challenge II: Scaling research in time: what happens in the long-term?” section). Time is intrinsic to the very idea of sustainable development, as exemplified by the Brundtland definition and its emphasis on the tradeoffs between the needs of current and future generations (UNWCED 1987). Choices made today have consequences that last for decades, centuries, or longer. The call for proposals specifically highlighted the possibility that research funded under the call might have “long-term significance” (Swedish Research Council Vetenskapsrådet Formas and Sida 2016). But research projects are short-lived. Each project in this study, for instance, was initially funded for a maximum of 3 years. An intrinsic challenge is how to resolve the tension between the short time horizon of research projects and the longer time horizons over which impacts may play out. Because research findings often only emerge toward the end of research projects, any knowledge application must take place after the time horizon of the research project itself. This interacts with the preceding challenge both because building an effective collaboration between researchers and knowledge users takes time and because funding for engagement with knowledge users often ends just as the results of multi-year projects become available.

The third challenge relates to *space* (“Challenge III: Scaling research in space” section). Space is fundamental to all environmental problems, whether via climate systems, the flow of surface water across different topographies, or through the spatial structure of ecosystems and living environments. Individual research projects have necessarily spatially constrained scales but attempt to draw lessons that can be extrapolated to different spatial scales—regional, national, or even global—or adapted for application to new contexts. This creates questions about scaling and validity of results, methods, and approaches to knowledge user engagement outside the original area of investigation.

In this paper we expand upon these three challenges, drawing on the ten research projects as case studies. Identifying these three challenges in turn suggests some practical pathways that might help overcome these challenges (“Outlook: Fixing the broken links?” section).

This perspective paper complements other studies of sustainability research. Many of these focus either on *what* has been researched in the past and *what* we should research in the future (e.g., Bhamra et al. 2011; Köhler et al. 2019; Clark and Harley 2020) or *how* we should go about it (e.g., Lang et al. 2012; Freeth and Caniglia 2020; Bentz et al. 2022). In this perspective, we contribute instead to the literature that focuses specifically on the translation of sustainability research into choices that shape impact (Cash et al. 2003; Clark et al. 2016b). Although some previous studies have highlighted one or more of the challenges we describe (Lemos and Morehouse 2005; Cash et al. 2006; Cornell et al. 2013; Polk 2014; Bansard et al. 2019; Knapp et al. 2019; Schäfer et al. 2020; Chambers et al. 2021; Balzarini et al. 2022), most previous studies focus on more narrowly or selectively defined groups of case studies (Cash et al. 2003; Van Kerkhoff and Lebel 2006; Clark et al. 2016a; Belcher et al. 2019; Wyborn et al. 2019; Jagannathan et al. 2020; Chambers et al. 2021) or had lower response rates (Hegger and Dieperink 2015; Newig et al. 2019). In contrast, this perspective covers an entire cohort of funded projects. This has two main advantages. First, the diversity of the projects included is arguably more representative of the full diversity of sustainability and resilience research, albeit as interpreted in a specific call for proposals. Second, we do not select projects based on whether or not they led to a particular outcome, such as a published paper, allowing us to fill a knowledge gap highlighted in previous research (Wyborn et al. 2019). These two features allow us to evaluate commonalities in experiences that transcend disciplines, specific approaches to knowledge user engagement, or particular project outcomes. Additionally, our perspective focuses on research projects in the Global South, historically understudied in research on sustainability (Moallemi et al. 2021) and in particular on the transition from research to impact (Clark et al. 2016a).

## Challenge I: Knowledge production to knowledge application

A successful transition from knowledge production to knowledge application, or use, requires several key steps. First, and perhaps so obviously that it is not always stated explicitly, knowledge that is produced must be useful, i.e., it must allow people to make better choices given the constraints that they face. Cash et al. (2003) call this property “salience” or relevance to decision-makers. To be useful, knowledge must also be both correct and perceived to be correct (or “credible,” Cash et al. 2003). Second, potential users must be aware of and understand this new knowledge and its potential benefits. This implies that knowledge must be in a format that its intended users can straightforwardly understand (Simis et al. 2016; Fløttum and Gjerstad 2017; McCall et al. 2017; Blake et al. 2018). It may also require that the knowledge production process be perceived as “legitimate,” or unbiased, respectful, and fair (Cash et al. 2003). Third, potential users must be able to apply and use this new knowledge, which in some cases may require further capacity building.

Researchers have hypothesized that a successful transition to knowledge application requires engagement with potential knowledge users at all stages of the research process. Engagement with potential users can in principle guide research toward usefulness, bring local insights (Dilling and Lemos 2011; Liguori et al. 2021), and help build a common language to describe problems and solutions. Some researchers go further and propose that potential users should collaborate and participate in designing research, generating knowledge and drawing conclusions (Baan and Klijn 2010), sometimes called co-production or co-creation of knowledge (Mauser et al. 2013; Schneider et al. 2021). The concept of knowledge co-production dates to the late 1970s and a policy agenda to re-orient the relationship between clients and providers in public service provision (Pestoff 2014; Sorrentino et al. 2018). Within academia it took root in at least three different fields: public administration; science and technology studies, and sustainability science (Miller and Wyborn 2020). The simultaneous progression toward knowledge production as a means to deliver “impact” in society boosted the popularity of co-productive approaches, however, it also made measuring and quantifying that very same impact much harder (Rau et al. 2018).

All projects in this portfolio involved engagement with potential end users of knowledge –ranging from waste pickers to international organizations–despite the constraints on travel and face-to-face contact during the COVID-19 pandemic. Many of the projects took innovative approaches. For example, project A lays out a novel approach to knowledge sharing about decentralized approaches to sanitation, using a collaborative serious game (RECLAIM, 1), a learning system that aims to educate while engaging and motivating participants (Billger et al. 2020; Kain et al. 2021). Project B seeks to close the gap between top-down and bottom-up design approaches for interventions to improve resilience to flooding in informal settlements, using a community responsive adaptation (CRA) approach that integrates community-level innovations with connections to technical expertise and wider governance systems (Mulligan et al. 2016). Project C facilitates international grassroots network connections between waste picker communities in five countries to promote innovation and knowledge dissemination (Zapata Campos et al. 2020). Project D develops a participatory scenario building tool that helps build consensus about land use transformation pathways among stakeholders in rural Kenya and Tanzania (Kariuki et al. 2021).

Despite these innovations, the consensus experience from the projects is that integrating research user participation into the research process is in practice far from straightforward, especially when the community of potential knowledge users is diverse, and that user participation does not remove all the constraints to knowledge use. Decision-makers may be locked in to paths shaped by earlier decisions, as in project A, where the majority of public investments still focus on historically conventional centralized approaches to infrastructure design despite their limited previous success (McConville et al. 2022). Political turbulence can exacerbate the short-termism created by election cycles and hamper the development of researcher-policymaker relationships, as project B encountered in Nairobi, Kenya. A perpetual challenge is to integrate bottom-up innovation with top-down governance structures, as in project C, where municipal officers and politicians perceive low-tech, locally adapted innovations developed by grassroots organizations as problems rather than solutions, preferring large-scale technological solutions. There may be multiple competing demands on stakeholders’ attention and time, as in project E, where efforts to promote biochar as a soil amendment are hampered by the difficulty of demonstrating benefits with long lead-times and complex causalities (Fridahl et al. 2021; Hansson et al. 2021; Rogers et al. 2022).

These experiences echo some other findings from previous literature describing the potential pitfalls or limitations of knowledge user participation in the research process (Van Kerkhoff and Lebel 2006; Oliver et al. 2019). Potential users may not always be immediately welcoming to information presented through science, for example because of fear of change, skepticism, or competition for funding and resources (Lang et al. 2012; Wehn et al. 2015; Gascoigne et al. 2020). Different user-groups may have different perceptions (Santoro et al. 2019), conflicting values (Afshar et al. 2016), or unequal internal power structures meaning that not all voices are heard (Agrawal and Gibson 1999; Arora-Jonsson 2014; Jiménez et al. 2018). Castán Broto and Neves Alves (2018) have also shown that policy-makers, communities, and scientists may have different objectives with the process of co-production, even where there is wide participation.

Previous contributions also highlight the potential for “boundary work,” or engagement specifically with communities of decision-makers to facilitate the transition from knowledge production to knowledge application (Cash et al. 2003). Three projects (B, C, and H) had formal non-academic project partners, and most engaged deeply with other non-academic actors, from local governments to international organizations.

The patterns we observe across the ten projects we study demonstrate that, in practice, knowledge user engagement alone cannot resolve the perennial challenge of transitioning from knowledge production into knowledge use. User engagement also adds new challenges relating to the dynamics of collaboration, capacity of users, and local power structures, for which the research community is not always well prepared. User engagement surely remains critical, not only for its potential instrumental value but also its intrinsic value in democratizing the research process. But even among a group of research projects that prioritizes end-user participation throughout the research process, barriers to knowledge application persist.

## Challenge II: Scaling research in time: What happens in the long-term?

**Fig. 4:**
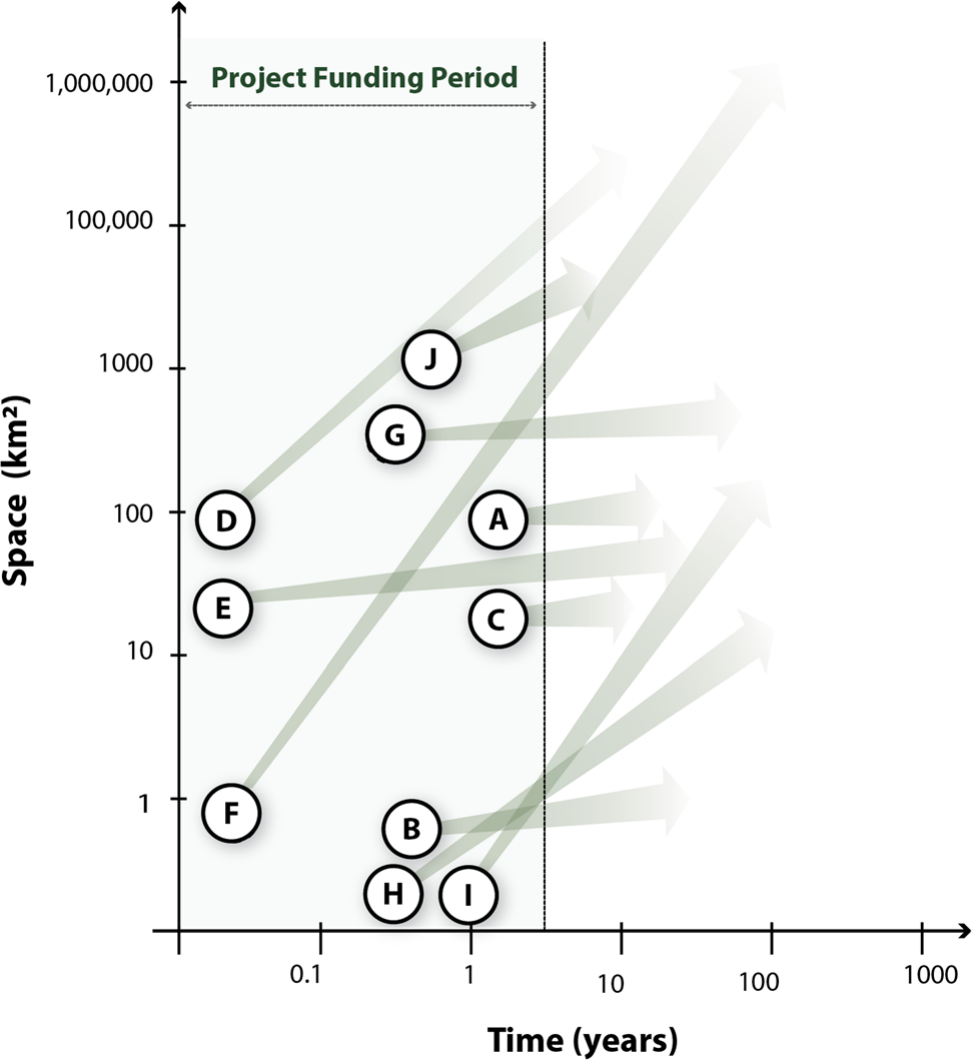
Summary of spatial and temporal scale of the projects and their intended impacts. White dots show approximate geographical temporal and spatial scale of research projects, while arrows point to temporal and spatial scales of intended impact. Space and time are shown in log scale

Sustainability and resilience are goals that necessarily must be approached with a long-term view, as the intergenerational view of sustainable development exemplified by the Brundtland definition makes clear. Our choices today have implications for many decades or centuries into the future, and choices made decades or centuries in the past still shape our lives today. These long-term goals contrast starkly with the short-term perspective driven by the 3- to 4-year funding cycle of most research projects, including those in this portfolio (Fig. 4).

The projects in this portfolio exemplify this tension in several respects. The tension originates with the mismatch between the time horizons of research projects and the time horizons intrinsic to the interventions and processes that sustainability and resilience researchers study. Interventions may themselves take several years to implement. For example, the intervention studied in project B, designed to improve flood resilience in informal settlements, takes around a year to design and implement at a single site, incorporating as it does an extended participatory design process. Further, the effects of interventions or changes in conditions may materialize over long time frames, for example, when they affect soil processes, as in projects D, E, F, and J, or the establishment of perennial crops, as in project F (Crews et al. 2018; Isgren et al. 2020). Projects that study climate change face a particularly salient challenge—climatic changes develop over decades or longer (see, e.g., projects G and H) (Von Uexkull and Buhaug 2021; Leizeaga et al. 2022). Such projects must necessarily find creative and innovative solutions to this problem: exploiting climatic variation across space to substitute for variation across time (space-for-time substitution, see projects E and H); using archaeological and paleo-ecological methods to generate proxy data about past social and environmental conditions (project D); or extrapolating from measured responses to short-term weather fluctuations to anticipate responses to changes in average climate (projects F and I). Lastly, projects that focus on resilience concern themselves with shocks that by definition occur infrequently (e.g., floods in project B, climatic fluctuations in project F), meaning that prospective studies need longer time horizons to observe responses to a distribution of shocks. The consequence is that the impacts of interventions or changes in conditions may only be measurable after the time frame of the initial research project.

This aspect of the problem of time also interacts with the previous challenge. The lessons we draw from these short-term research projects are intended to influence the choices of individuals and policy-makers over still-longer time horizons, and these choices in turn have consequences that play out over time (Fig. 4). An extreme example of this comes from project D, which concludes that time frames of hundreds of years are necessary to understand why and how present day conditions in the Serengeti basin arose, how people responded to previous climate challenges, and thus how to plan future land use (Courtney Mustaphi et al. 2019). However, funding for interaction with users is limited to the project’s time horizon. Funding for user interaction is thus only available when a research project is already funded, often implying that the research question must be fixed before extensive engagement with users can take place and undermining the possibilities for genuine co-creation of knowledge. In practice, most of the projects we study build on previous research and established relationships with stakeholder organizations, and some build in forward-looking workshops with knowledge users at the close of this funding cycle to inform future research (project I). Funding may also be cut off just as results from longer-term research projects are becoming available. For example, in a project designed to understand how de-intensification of agriculture can improve resilience and sustainability of croplands (project H) (Leizeaga et al. 2022), researchers built up interest in research findings through regular participation in annual meetings with farmers and other land users, but funding ended just as the project was at the cusp of translating knowledge into practitioner use.

The result of this mismatch between the timing of funding for user engagement and the emergence of results may be piecemeal implementation of lessons from research that is no longer firmly based on the actual science. The challenge compounds the fundamental constraint that policymakers want answers immediately, but research takes time and is further complicated by political decision-making cycles that incentivize quick wins over long-run social goals and result in turnover in political actors and allies (project B).

The time problem also suggests a fundamental limitation to the approach of co-creating or co-producing knowledge with potential users in sustainability research. Sustainability problems are intrinsically intergenerational, and knowledge we produce today may inform the choices of future generations as well as current generations, but these future knowledge users can never be engaged in the knowledge production process.

## Challenge III: Scaling research in space

Whether or not we achieve global development goals depends on coordinated action across all countries and regions of the world. Space is thus intrinsic to understanding environmental sustainability and resilience. The contexts in which these projects take place illustrate these spatial interdependencies. Flood risk reduction interventions taken in one area affect flood risk in others (project B), just as upstream forest restoration (project I) or water management systems (project J) affect downstream water availability. Global climatic change increases the risks of environmental hazards worldwide, potentially exacerbating the risk of conflict in fragile contexts (project G).

The projects that we study aim to influence the choices of individuals or policymakers over a wider spatial scale than that of the research itself (see Fig. 3). The consistency of these aims is itself noteworthy, given that previous literature debates over to what extent and over which scales knowledge can be transferred and to what extent co-produced knowledge can serve other potential users not directly engaged with co-production of knowledge (Merton 1973; Polk 2014; Sutherland et al. 2017; Nagy et al. 2020; Lu et al. 2022). While few among the projects in this study aspired to global applicability, all hoped to generate knowledge applicable to scales beyond the project itself. This creates challenges related to scale-specific solutions: something that works at one scale may not work at a different scale or in a different place.

The first type of spatial challenge relates to upscaling of results. Increasing spatial scale may alter the outcome of a process or intervention. For example, processes investigated at a wider scale might follow different ‘rules’ compared with the same processes observed at smaller scales, because as the spatial scale widens, the context in which processes occur becomes more heterogeneous (Chesson 2012). Soil properties can change in response to land management at a local scale, but the same effect might not be apparent at a larger scale because land management interacts with other environmental conditions in such a way that its ‘average’ effect becomes negligible. This requires upscaling or downscaling (Chesson 2012) to extrapolate from small-scale data to large-scale predictions or vice versa. For example, project I combines data-based (empirical) and process-based (mechanistic) modeling approaches to scale up forest biogeochemical processes in space and thus identify areas most suitable for forest restoration in Cambodia (Johansson et al. 2020; Venkatappa et al. 2020). Project J studies the relationship between agricultural intensification and rice yields in the Mekong Delta, Vietnam, using spatial sampling to ensure representativeness with respect to the patchwork of local land and water management strategies (Livsey et al. 2021). Interventions that promote a new crop or technology may not be as effective at larger scale if widespread adoption increases supply and causes prices to fall (Janvry et al. 2017; Burke et al. 2019) or because other players become important and alter the outcome of the intervention. This type of scaling is particularly important because policy is often designed not at a fine-grained local scale, but by or across nations (Dearing et al. 2014; Steffen et al. 2015).

The second type of spatial challenge is that knowledge generated at one place may not be fully applicable in different contexts. Even more important than knowing *what works* may be knowing *where else* it will work and why. Climatic conditions, ecosystems, institutions, and stakeholders needs and aspirations all vary across space. There may be differences in local technical capacity that affect the ability to adopt innovations, such as the technical capacity required to operate and maintain pyrolizers that produce biochar (project E) or pathogen ecologies that affect whether new crop varieties thrive (project F).

Linkages between existing research projects can help address this challenge. For example, researchers in project F collaborated with existing long-term plant breeding programs in the US and China, first obtaining promising cultivars for testing in Uganda and later feeding back results to inform future breeding, specifically of cultivars that may be less susceptible to pathogens.

Space also interacts with the first challenge, the transition to knowledge application. Knowledge that has been created in one region is likely to be more applicable to potential users in neighboring regions than farther afield, especially when the neighboring regions share social, climatic, and ecological conditions (Diamond, 1997). Knowledge about successful local precedents may spread more easily across space if spatial proximity allows for in-person learning from previous successful examples, as in project E.

Experience with the ten research projects thus reveals a tension: almost all projects aspired to create knowledge that is applicable, if not universally, then at least across broader spatial scales than that on which it was generated. But all reported challenges in doing so. Which of these challenges can be overcome, and which are intrinsic limitations on the transfer of knowledge across space, remains to be determined.

## Outlook: Fixing the broken links?

Dedicated funding for sustainability and resilience research, as exemplified by the call for proposals that funded these projects, is essential if we are to generate the knowledge and innovation required to meet our collective social goals. The sustainability and resilience research projects reviewed in this perspective have resulted in long-lasting collaborations not only between researchers from Sweden and the Global South but also with knowledge user communities (Table 1), facilitated by the structure of the call for proposals. However, reflecting on these projects at the end of the funding cycle has led us to identify some “broken links” that impede the translation of research on sustainability and resilience into real-world impact. The first relates to the transition from knowledge production to knowledge application (“Challenge II: Scaling research in time: what happens in the long-term?” section); the second relates to the tension between the short-run time horizon of research projects and funding calls, and the often longer-run time-horizons of social goals and the underlying environmental processes we study (“Challenge III: Scaling research in space” section); and the third to the extrapolation of research findings across space (“Challenge III: Scaling research in space” section). These challenges are common across sustainability and resilience research projects in diverse disciplines and contexts across the Global South. The challenges persist despite top-down funder commitment to promoting North–South collaboration, and despite researcher commitment to the goals of translating research into real-world impact and to the practice of engaging potential knowledge users in the research process.

One may reasonably ask whether the project is the right unit of observation to study the research process and whether the end of the funding cycle is the right time to evaluate the transition from knowledge production to impact. Indeed, most of the projects have sought or are actively seeking follow-up funding, although fewer than half of the projects in the portfolio have secured it. Indeed, an implication of the time challenge is that impact would be better evaluated years after the end of the funding cycle, as others have proposed (e.g., Cash et al. 2003; Newig et al. 2019). Nonetheless, given the status quo, we believe it remains valuable to record our observations about this set of research projects at the end of the funding cycle. In the future, we plan to revisit and update the conclusions we draw in this perspective.

How might we fix these “broken links”? Across ten projects so diverse in disciplines, scopes, scales, and contexts, the ubiquity with which projects encountered the three challenges suggests that “fixing” these links might not be straightforward. Building long-term, scalable pathways to sustainability and resilience may require changes that go beyond the decisions and efforts of individual researchers or research teams (see also Schneider et al. 2019; Wyborn et al. 2019; Otero et al. 2020). Reflecting on these challenges does, however, suggest some potential avenues for progress.

First, how might we more effectively translate research into real-world impact? Our experiences suggest that barriers to this transition persist, even when projects prioritize user engagement throughout the research process. Reviewing the literature reveals an abundance of potential strategies that researchers might apply. Several scholars point to the need to acknowledge power structures (Castán Broto and Neves Alves 2018; Speckhahn and Isgren 2019), and others emphasize actor representativity and the nesting of projects into larger decision-making structures (Farr 2018; Miller and Wyborn 2020). However, understanding which strategies are likely to be effective in which contexts is difficult because of a relative lack of evidence about *whether* and *when* these strategies work, and how to standardize measurement of the real-world impact of research (Lemos et al. 2018). Research funders might consider directly funding targeted research on how to systematically measure impact and how to evaluate these strategies (see, e.g., Oberlack et al. 2019; Belcher et al. 2020). A precedent is a nascent literature which uses experiments to evaluate how different approaches to participation shape decision-making and project impacts (Madajewicz et al. 2021; Grillos 2022). Changing incentives for researchers, including promotion and recruitment, to more directly reward social impact might also help drive innovation and experimentation, as opposed to the current system, which primarily rewards publications and research funding, and neglects whether or not research outputs translate into real-world impact. Therefore, we note with interest the recent initiative by Science Europe—with support from the European Commission—to re-orient assessment practice within academia away from narrow bibliometric approaches (Science Europe 2022). But we caution that such a change in incentives might not be straightforward. Time frames for real-world impact (Cash et al. 2003; Schneider et al. 2019; Belcher et al. 2020) or effective collaboration-building with knowledge user communities (Armitage et al. 2011; Lux et al. 2019) are longer than those for publication of results from short-term projects. Increasing incentives for impact without considering this time horizon or resolving the problem of how to systematically measure impact is unlikely to increase real-world impact.

Second, to address the disconnect between research project time horizons and impact time frames, funders might allocate specific funds for post-project engagement, dissemination, outreach, and implementation (see also Schneider et al. 2019). A real-world example is a new funding model currently being piloted by the International Science Council “Regional Sustainability Hubs,” which aim to fund and facilitate stakeholder engagement in all stages of the research process (International Science Council 2023). More generally, additional funding could be limited to teams that have successfully completed funded research projects within the last 5 years, allowing successful collaborations to build on already-established trust between researchers from different backgrounds and user communities. Funders could also provide grants to early-career researchers that are specifically directed to funding engagement with knowledge users, with a view to allowing junior researchers to establish collaborations and develop research proposals, or organize matchmaking events that pair researchers with potential partner organizations. Funders could also more generally support projects over longer time horizons. However, given finite budgets for research funding, this would almost certainly lead to a trade-off between few longer-duration projects and many shorter-duration projects.

Finally, to address the problems of scaling up research findings across space, funders might consider how to promote connections between researchers, projects, or local organizations working on related or complementary issues (see, e.g., Bai et al. 2019; Norström et al. 2022; Barraclough et al. 2023; Future Earth 2023) or even to directly facilitate collaboration and coordination across different sites (see, e.g., Slough et al. 2021). New, remotely sensed spatial datasets also now map the world at unprecedented resolution and scale, for example, project I used forest cover data at 30-m resolution, but modern products map forests almost to the resolution of single trees with up to daily temporal resolution (von Carnap 2022; Reiner et al. 2022; Wagner et al. 2023). These new data products are revolutionizing our ability to quantify heterogeneities in the biophysical and socioeconomic environments (Burke et al. 2021), supporting spatial scaling efforts in at least some cases. These innovations help illustrate the essential role of research infrastructure in underpinning research in sustainability and resilience.

The ultimate goal driving researchers, funders, and user communities alike is a sustainable future for our planet. While we have focused here on documenting challenges that emerge in translating sustainability and resilience research into sustainable development, we are not pessimistic. The process of systematically documenting these challenges has allowed us to formulate proposals for some systemic improvements and, we hope, paves the way for further innovation in the future.

### Supplementary Information

Below is the link to the electronic supplementary material.

Supplementary file1 (PDF 279 KB)

## Footnotes

1 https://www.slu.se/reclaim.
