# Supplementary material for: Pathways from research to sustainable development: Insights from ten research projects in sustainability and resilience
Source: Ambio. 2024 Feb 7;53(4):517–33. doi: 10.1007/s13280-023-01968-4 (PMC10920586; doi:10.1007/s13280-023-01968-4)
Supplement: Supplementary file 1 — Supplementary file1 (PDF 279 KB) [file 13280_2023_1968_MOESM1_ESM.pdf]

Ambio

Electronic supplementary material

This supplementary material has not been peer reviewed.

Title: “**Pathways from research to sustainable development: insights from ten research projects in sustainability and resilience”**

Anna Scaini, Joseph Mulligan, Håkan Berg, Albert Brangarí, Vera Bukachi, Sebastian Carenzo, Chau Thi Da, Colin Courtney-Mustaphi, Anneli Ekblom, Hanne Fjelde, Mathias Fridahl, Anders Hansson, Lettice Hicks, Mattias Höjer, Benard Juma, Jaan-Henrik Kain, Rebecca W. Kariuki, Soben Kim, Paul Lane, Ainara Leizeaga, Regina Lindborg, John Livsey, Steve W. Lyon, Rob Marchant, Jennifer R. McConville, Linus Munishi, David Nilsson, Luke Olang, Stefan Olin, Lennart Olsson, Peter Msumali Rogers, Johannes Rousk, Hans Sandén, Nophea Sasaki, Anna Shoemaker, Benjamin Smith, Lan Thai Huynh Phuong, Ana Varela Varela, Manjunatha Venkatappa, Giulia Vico, Nina Von Uexkull, Christine Wamsler, Menale Wondie, Patrick Zapata, María José Zapata Campos, Stefano Manzoni, Anna Tompsett

Summary of the ten projects

This section provides further details on the research projects used in the main text to exemplify the challenges of sustainability and resilience research. The project characteristics include:

- type of partnerships, and whether these partnerships were stated in the proposal (formal) or developed in the project (informal);
- disciplinary background of the researchers involved;
- non-academic project partners, if relevant, e.g., NGOs, policymakers, local government bodies;
- knowledge users/stakeholder involved, e.g., local administrators, farmers;
- role of researchers and non-academic partners, e.g., data analysis, communication, implementation, data collection;
- plan for project continuation, if any;
- qualitative description of challenges encountered.

Project A. Adaptation and innovation in sanitation planning (Kampala, Uganda)

Full title: Adaptation and innovation in sanitation planning: exploring technical and societal readiness of alternative nutrient-recovery systems

Type of partnerships: Formal with academic project partners. Informal partnerships were developed in the project with Ugandan academic partners, municipal actors and NGOs.

Researchers' Background: Civil & Environmental Engineering, Biology, Architecture.

Non-academic project partners: None formally involved, but local NGOs, utilities and government workers were involved in the co-design of the serious game, as well as policy briefs and one scientific publication.

Knowledge users/stakeholders involved: Engaged actively the water utility providers, the Minister for Water, National agricultural research center and NGOs in the development of the serious game, policy briefs and interpretation of the results.

Role of researchers: Project design, data collection, data analysis, writing academic outputs, facilitating co-design process with serious game, disseminating research findings to communities.

Roles of non-academic partners: Sources of knowledge, data collection, data validation & analysis, participation in co-design process, review of policy briefs.

Plan for project continuation: A communication project has been funded to continue development and distribution of the serious game. Several proposals have been submitted.

Challenges: In the sanitation sector, innovations are hampered by both a lack of knowledge about new options and a tradition of large investments in centralized sewage infrastructure that only serves a fraction of urban dwellers. This project addresses this issue by investigating how to scale-up nutrient-recovery from wastewater in urban areas in Uganda, as a means to increase the resilience of sanitation systems in the face of environmental changes. Despite the fact that only 1% of Ugandans are served by centralized sewage, the majority of sanitation-related university courses and public investments focus on this

infrastructure. This underlines the need for improved communication and knowledge dissemination to promote alternative, and more sustainable, solutions. To increase collaboration between stakeholders and raise awareness of resource recovery opportunities in sanitation, the research team developed a collaborative *serious game* (RECLAIM, https://www.slu.se/reclaim), a learning tool that aims to educate while engaging and motivating participants. Initial tests with the game in Kampala demonstrated that stakeholders acquired new knowledge about resource recovery from sanitation, were more open to dialogue, and that the game also stimulated new ideas. In addition, a technical guide to sanitation technologies for resource recovery was published as a means to easily disseminate knowledge on alternative solutions. However, changing university curriculum and public infrastructure takes time and it is difficult to see direct impacts from such knowledge sharing and collaborative approach over a short time period. Indeed, given the diverse factors that affect decision-making it is difficult to establish causal relationships between knowledge and action.

Key references: Billger et al. (2020); McConville et al. (2020); Kain et al. (2021); McConville et al. (2022).

Project B. Community-responsive adaptation to flooding in informal settlements (Nairobi, Kenya)

Full title: Flood resilience in slums: community-responsive adaptation in Kibera, Nairobi

Type of partnerships: Formal partnerships between academic partners and practice organization in Kenya, originating from proposal development. Multiple informal partners to deliver interventions

Researchers' Background: Economics, Civil Engineering, Hydrology, Urban Planning.

Non-academic project partners: NGOs, Community Based Organizations (CBOs) formally engaged. Local government engaged in permissions and in advocacy/dissemination activities.

Knowledge users/stakeholders involved: Residents of informal settlements, practitioners (architects, engineers, landscape architects, planners), local government.

Role of researchers: Project design, evaluation design, data analysis, writing academic outputs.

Roles of non-academic partners: Intervention design/delivery, data collection, advocacy, contributing to academic outputs.

Plan for project continuation: Analysis, report-writing and advocacy continuing unfunded. Planning to submit proposals for future continuation.

Challenges: Flooding in informal settlements is a major global concern because informal settlements are often located in flood-prone areas. Top-down approaches to these problems have not historically been successful because government actors have limited ability to act effectively in these areas, while bottom-up solutions based on individual initiatives cannot solve these watershed-scale problems. This project developed a community responsive adaptation (CRA) approach to complement community-level innovations with connections to technical expertise and wider governance systems, with a focus on Kibera, Nairobi. Panel data for close to 1500 households tracked flood impacts over time and space, as well as residents' experiences of the CRA process. The project team engaged local administrators and Members of Parliament. The emerging results support the case - from both cost and impact perspectives - for a community-informed approach. However, ongoing changes in the municipal governance system are

creating political turbulence, which exacerbates the short-termism of election cycles and hampers the development of researcher-policymaker relationships that can influence policy and practice.

Key references: Mulligan et al. (2016;) Mulligan et al. (2019); Juma et al. (2021); Wamsler et al. (2022).

Project C. Grassroots approaches for climate, environmental, and poverty challenges in recycling networks (multi-site)

Full title: Recycling networks. Grassroots resilience tackling climate, environmental and poverty challenges

Type of partnerships: Formal partnerships between universities, grassroots organizations (waste pickers organizations and networks), and local governments. During project implementation new coalitions were established with e.g. WIEGO (Women in the Informal Economy Global Organization), RedLacre (Latin American Network of Waste Pickers), UN-Habitat.

Researchers' Background: Anthropology, sociology, water and sanitation engineering, urban planning, human geography, public administration, business administration, political science

Non-academic project partners: NGOs, CBOs, grassroots organizations (resident associations, waste pickers associations), local government.

Knowledge users/stakeholders involved: Waste pickers, waste picker organizations and networks, residents of informal settlements and resident associations, municipal officers and politicians working with waste management, NGOs supporting inclusive &. sustainable waste governance.

Role of researchers: Project design, data collection, data analysis, writing academic papers, producing policy briefs and videos for communication, conference organization, facilitating a peer-to-peer transfer technology workshop.

Roles of non-academic partners: Data collection (waste picker organizations got involved in design and collection). We also met in two international meetings to discuss preliminary results together, policy advocacy during conferences.

Plan for project continuation: Obtained a Formas project for communicating research results, we have produced new videos with innovations and policy briefs, and organized several international waste picker conferences in Kenya and Tanzania (2021-2022). Plan to submit new proposals.

Challenges: Grassroots organizations and networks face challenges when attempting to build up a participatory waste governance for more inclusive and sustainable cities. Informal waste pickers are increasingly connecting with each other through local, regional and global networks. Unlike the standardized—but difficult to apply—knowledge generated by donors and international organizations, these networks disseminate locally-developed, innovative, and flexible solutions. This project enabled meetings between waste pickers, researchers, community-based organizations, county and city officers and politicians, that sparked peer to peer knowledge exchanges between waste picker organizations from different countries (Kenya, Tanzania, Argentina, Brazil, Nicaragua). These interactions led to the design, fabrication and testing of low-tech, locally-adapted innovations (a push cart, a manual press, and a biogas digestor), demonstrating that low-tech and low-cost solutions can be developed when grassroots organizations are given freedom to innovate according to their contextual problems and resources. However, these innovations are often perceived as a problem rather than as a solution by municipal officers and politicians, who instead prefer large-scale technological solutions. This perception, together with structural problems of corruption, lack of resources for waste management, devolution of power, and economic crises put many of the grassroots innovations at risk of disappearing.

Key reference: Zapata Campos et al. (2020); Zapata Campos et al. (2023c); Zapata Campos et al. (2023b); Zapata Campos et al. (2023a)

Project D. Lessons from the past for adaptation and resilience to climate change (Kenya, Tanzania)

Full title: Adaptation & resilience to climate change

Type of partnerships: Formal partnerships with university-based researchers. These were extended to include other academic partners in the region, alongside various informal links and collaborations with local stakeholders, including CBOs, NGOs, local government departments.

Researchers' Background: Archaeology, Palaeoecology, Ecology, History, Geography, Environmental Sciences.

Non-academic project partners: None formally involved.

Knowledge users/stakeholders involved: Pastoralists, farmers, hunter-gatherers, wildlife managers, CBOs, NGOs, local government officers, tourism operators, heritage professionals, faith-based leaders, land use planners, socio-economic researchers, environmentalists.

Role of researchers: Project design, evaluation design, data analysis, writing academic outputs, facilitating knowledge co-production workshops, disseminating research findings to communities.

Roles of non-academic partners: Knowledge co-production workshops, data sharing, dissemination, contributions to academic publications.

Plan for project continuation: No additional funding secured, although several grant applications submitted; new applications pending; academic writing and data analysis continue unfunded.

Challenges: Planning for adaptive and resilient responses to climate change must be founded on a sound understanding of the past environmental conditions, socio-cultural, economic, regulatory, and political circumstances, and the spatial distribution of human activities and land uses. By applying a participatory scenario tool (KESHO – a Swahili word meaning "tomorrow"), stakeholder understanding of past and visions for future land use were brought together, to define the key drivers of land use transitions and how these have shaped the landscape today and are likely to evolve in the future. This approach is challenging first because it is not clear how far back in the past we need to extend our knowledge to understand

present-day conditions and people´s capacity to adapt in the future. By focusing on the last 300 to 500 years, this project captured considerable changes to socio-cultural and environmental landscapes that could be used for planning sustainable and resilient futures. The second challenge is how far into the future we can and should plan for change. Here, the target dates for the SDG and African Union's Development Agenda—2030 and 2063 respectively—were selected. Using the KESHO tool, stakeholder visions for future land use were combined with spatial data, personal memories and lived experiences, each leading to four divergent land use futures in 2030 and 2063. These plausible futures can help stakeholders, communities and policy developers guide interventions and planning, including changes in practices and policy, to achieve a desirable and sustainable future, or at least avoid undesirable ones.

Key references: Courtney Mustaphi et al. (2019); Kariuki et al. (2021).

Project E. Trade-offs in biochar production and use (Tanzania)

Full title: Conflicting ambitions concerning the use of biomass: Sweden, Tanzania and the world

Type of partnerships: formal with academic project partners, informal with local stakeholders that was not stated in proposal but developed during the project's duration.

Researchers' Background: social science (sociology, technology and social change, environmental change), agriculture science, soil science, botany.

Non-academic project partners: None formally involved

Knowledge users/stakeholders involved: Farmers, local administrators, local NGOs

Role of researchers: Project design, evaluation design, data analysis, writing academic outputs, outreach

Roles of non-academic partners: Data collection, hosting workshops, dissemination

Plan for project continuation: Formas Grant in 2019 (2019-2024) that allows to continue the collaboration and expand the empirical scope. The new grant is explicitly a continuation of the VR project. Currently no plans to submit additional applications.

Challenges: Biochar can contribute to both agricultural productivity and atmospheric carbon dioxide removal. In contrast to large-scale and technically advanced bioenergy with carbon capture and storage, biochar is low-tech and small-scale, aligning local needs with global aspirations to mitigate climate change. However, enabling to sustain action over time emerges as a central concern for biochar. Through local field studies in the Karagwe, Mbeya, and Songwe regions (Tanzania), and a synthesis of Tanzanian biochar projects, we have identified several time-related causes of failure of these efforts. These include the difficulty of demonstrating benefits with long lead-times and complex causalities that often involve conflicts with local subsistence strategies or trade-offs between aspirations (e.g., using biochar as a cooking fuel instead of a soil amendment). Adapting external operational skills to local contexts has also proven challenging, as has securing long-term financing for maintenance of the pyrolizers. Experiences from research projects and farmers' own use of biochar showed that global or regional benefits need to be

demonstrated without compromising local needs. We identified successful projects and supported their dissemination to neighboring communities, arguably a necessity for scaling up and sustaining biochar projects over time.

Key references: Fridahl et al. (2021); Hansson et al. (2021); Rogers et al. (2022).

Project F. Multifunctional perennial crops for increased durability and resilience (Uganda)

Full title: Development and adoption of multifunctional perennial farming systems: towards increased sustainability and resilience

Type of partnerships: Formal partnerships with university and national agricultural research organization. Collaboration agreements with 10 small groups of farmers to test and document standardized trials of perennial crops. Financial compensation for land and labor.

Researchers' Background: Human ecology, human geography, sustainability science, physical geography.

Non-academic project partners: None formally involved.

Knowledge users/stakeholders involved: Farmers and Rural Advisory Services.

Role of researchers: Project design, evaluation design, data analysis, writing academic outputs, outreach.

Roles of non-academic partners: Source of knowledge, carrying out trials, reporting on trials

Plan for project continuation: Agreement signed for continued breeding of sorghum for five years, to be funded by a project partner in USA. Continued breeding and promotion of perennial rice in collaboration with a Chinese research partner brought in by the project.

Challenges: Domestication and plant breeding develop over several decades, so how can we conduct research on perennial crops that are potentially more resilient to climatic fluctuations than annual crops, in just three years? Promising sorghum and rice cultivars from the Land Institute (USA) and the International Joint Research Center for Perennial Crops (China) have been tested by farmers in Uganda. Rice was a success, but there are challenges to the idea of shifting to perennial cropping systems. In contrast, sorghum was susceptible to a fungus (ergot). This important result was useful to feed into the breeding of a new variety – hopefully less susceptible to ergot – that will continue to be tested beyond the time frame of this project. A general problem of perennial cropping systems is that they are often slower to establish than annual ones. To partly overcome this issue and maximize the chances of rapid

establishment of the perennial varieties, the participating farmer groups were spread geographically along a climate gradient from humid to sub-humid conditions. However, to reliably test the perennial varieties, a longer time frame, together with a wide range of growing conditions, is necessary. Importantly, the project established a research capacity for participatory breeding that will be funded through other channels for at least another 5 years.

Key references: Crews et al. (2018); Isgren et al. (2020).

Project G. Climate change, food security and armed conflict (global)

Full title: Climate change, food security and armed conflict

Type of partnerships: formal with academic project partners, informal with United Nations Food and Agriculture Organizations (FAO) for data collection and analysis.

Researchers' Background: peace and conflict research, sociology, anthropology.

Non-academic project partners: None formally involved.

Knowledge users/stakeholders involved: None formally involved.

Role of researchers: Project design, data collection, data analysis, writing academic papers, conference organization.

Roles of non-academic partners: -

Plan for project continuation: There is no funding to continue activities, but new projects that build on the insights gained in the project such as work in the Mistra Geopolitics research program funded by the Swedish Foundation for Strategic Environmental Research Mistra on food security.

Challenges: Assessing societal responses to long-term climate change is challenging because: i) societies may respond differently to long-term gradual climate changes than to short-term shocks, and ii) we extrapolate from patterns in current societies to the future. However, societal resilience to climate change can evolve in tandem with climate change, thus altering adverse impacts. In this project, we combined knowledge from multiple lines of evidence at individual, sub-national, national, and global scales, and identified patterns and pathways linking climate change, food security and armed conflict in current societies. We used this evidence to understand how societies respond to environmental shocks and under what conditions violent responses are likely. This evidence can inform the identification of potential future areas and populations at risk if societies fail to increase resilience to environmental hazards like droughts, floods and storms that will become even more likely and severe with increasing climate change.

Key reference: Von Uexkull and Buhaug (2021).

Project H. The resilience and sustainability of soil microbial functions to drought (Ethiopia)

Full title: The resilience and sustainability of soil microbial functions to climate change induced-drought in Ethiopia

Type of partnerships: Formal with academic project partners, and agricultural institutes, informal with stakeholders involved.

Researchers' Background: Ecology, Geosciences, Forestry, environmental science, agriculture.

Non-academic project partners: Local government research institute: Amhara Regional Agricultural Research Institute (ARARI).

Knowledge users/stakeholders involved: Practitioners (farmers, forestry practitioners) involved through data collection, guards of research infrastructure. Local government via ARARI. Regional farmers and forestry practitioners through organized annual meetings at ARARI.

Role of researchers: Project design, evaluation design, data analysis, writing academic outputs, outreach

Roles of non-academic partners: Data collection on field experimental plots, maintenance of research infrastructure, guards of infrastructure.

Plan for project continuation: We continue with minimal funding to maintain field experiments, and have applied for further funding.

Challenges: The study of soil responses to climate change requires a long-term perspective, which is challenged by a three-year research cycle. To overcome this challenge and assess nutrient provision and carbon storage in agricultural soils in the Amhara region (Ethiopia), geographical differences in climate were leveraged, enabling a 'space-for-time' substitution approach (e.g., Currie et al. 2021). Along the climate gradient from cool and moist highland sites to hot and dry lowland sites, further change was simulated by installing rain shelters to experimentally induce drought, and open-top chamber greenhouses to experimentally induce warming. This allowed to identify whether the simulated short-term drier and warmer conditions resulted in the same effects as those seen along the long-term climate gradient. Results

were shared with farmers, land owners and practitioners with help from the Amhara Regional Research Institute (ARARI), in a communication effort based on reciprocal trust developed over the duration of the project. Specifically, the project is informing farmers on how de-intensifying agriculture influences soil carbon sequestration, provision of nutrients for plant growth, and ecosystem functioning during drier conditions anticipated due to climate change. An ongoing challenge is finding resources to maintain the established field-experiments and to continue supporting knowledge-use by local stakeholders.

Key reference: Leizeaga et al. (2022).

Project I. Forest restoration and effects on water resources for smart agriculture (Cambodia)

Full title: Forest restoration and water availability for smart agriculture: a case study of Cambodia

Type of partnerships: Formal with academic project partners, informal with stakeholders involved.

Researchers' Background: Ecology, Geosciences, Forestry.

Non-academic project partners: None formally involved.

Knowledge users/stakeholders involved: National government agencies, local government and community organizations, NGOs.

Role of researchers: Field data collection, data analysis and development of tools, capacity building activities, stakeholder engagement activities.

Roles of non-academic partners: -

Plan for project continuation: No / applying for funding is under consideration to capitalize on capabilities and relationships developed during the project.

Challenges: How can we identify areas most suitable for forest restoration? Data-based (empirical) and process-based (mechanistic) modelling approaches were combined to scale up forest biogeochemical processes in space and time. Using the Google Earth Engine platform and informed by field data on carbon stocks, vegetation and land use, and local expert knowledge, we mapped forest cover change at 30-metre resolution over the last three decades, capturing the joint effects of climate and land use drivers. This data-based product was used to validate and inform a process-based vegetation and ecosystem model, which was then used to project future forest productivity, carbon stocks, and vulnerability to climate change and shocks. Together, these modelling approaches provide a basis to inform land use decisions, in this case the identification of suitable areas for forest restoration to sustainably manage carbon stocks, water resources and biodiversity under current and future climatic conditions.

Key references: Johansson et al. (2020); Venkatappa et al. (2020).

Project J. Trade-offs between ecosystem service provision and water management in rice systems (Vietnam)

Full title: Quantifying the trade-offs between ecosystem service provision and water management in rice systems

Type of partnerships: Formal with academic project partners, informal with stakeholders involved.

Researchers' Background: Engineering, hydrology, agriculture, environmental science.

Non-academic project partners: None formally involved.

Knowledge users/stakeholders involved: Farmers, local administrators.

Role of researchers: Data collection and analysis, communication to academic audience and stakeholders.

Roles of non-academic partners: Expert knowledge, data collection, communication.

Plan for project continuation: Applying for funding is under consideration.

Challenges: In the Mekong Delta (Vietnam), dykes and canals regulate flood water and allow growing three rice crops per year, but yields in the three-crop system are declining. Soil sampling across the An Giang province demonstrated that plant-available nutrients can be even higher in the three-crop compared to the two-crop system, suggesting that the former, more intense management is compromising ecological functions and promoting pests that negatively affect yields. This project focuses on one province in Vietnam with the goal of applying knowledge to other areas in SE Asia. Understanding how agricultural intensification affects rice yields is complicated by the patchwork of local land and water management strategies, requiring a spatial sampling strategy that ensures representativeness in the study area. Moreover, soil properties are partly controlled by larger scale processes (water flow in the Mekong River). This variability in space hinders applicability of results to broader contexts; regional to continental influences on the study area make implementation of management changes even more challenging.

Key reference: Livsey et al. (2021).

References

Billger, M., J. H. Kain, C. B. Niwagaba, and J. R. McConville. 2020. Lessons from co-designing a resource-recovery game for collaborative urban sanitation planning. *IOP Conference Series: Earth and Environmental Science* 588. doi:10.1088/1755-1315/588/4/042041.

Courtney Mustaphi, C. J., C. Capitani, O. Boles, R. Kariuki, R. Newman, L. Munishi, R. Marchant, and P. Lane. 2019. Integrating evidence of land use and land cover change for land management policy formulation along the Kenya-Tanzania borderlands. *Anthropocene* 28: 1–21. doi:10.1016/j.ancene.2019.100228.

Crews, T. E., W. Carton, and L. Olsson. 2018. Is the future of agriculture perennial? Imperatives and opportunities to reinvent agriculture by shifting from annual monocultures to perennial polycultures. *Global Sustainability* 1: 1–18. doi:10.1017/sus.2018.11.

Currie, T. E., M. Campenni, A. Flitton, T. Njagi, E. Ontiri, C. Perret, and L. Walker. 2021. The cultural evolution and ecology of institutions. *Philosophical Transactions of the Royal Society B: Biological Sciences* 376: 1–13. doi:10.1098/rstb.2020.0047.

Fridahl, M., S. Haikola, P. M. Rogers, and A. Hansson. 2021. Biochar Deployment Drivers and Barriers in Least Developed Countries. In *Handbook of Climate Change Management*, ed. W. Leal Filho, J. Luetz, and D. Ayal, 119–148. Springer. doi:https://doi.org/10.1007/978-3-030-22759-3_324-1.

Hansson, A., S. Haikola, M. Fridahl, P. Yanda, E. Mabhuye, and N. Pauline. 2021. Biochar as multi-purpose sustainable technology: experiences from projects in Tanzania. *Environment, Development and Sustainability* 23: 5182–5214. doi:10.1007/s10668-020-00809-8.

Isgren, E., E. Andersson, and W. Carton. 2020. New perennial grains in African smallholder agriculture from a farming systems perspective. A review. *Agronomy for Sustainable Development* 40: 1–14. doi:10.1007/s13593-020-0609-8.

Johansson, E., S. Olin, and J. Seaquist. 2020. Foreign demand for agricultural commodities drives virtual carbon exports from Cambodia. *Environment Research Letters* 15: 1–10. doi:10.1088/1748-9326/ab8157.

Juma, B., L. O. Olang, M. Hassan, S. Chasia, V. Bukachi, P. Shiundu, and J. Mulligan. 2021. Analysis of rainfall extremes in the Ngong River Basin of Kenya: Towards integrated urban flood risk management. *Physics and Chemistry of the Earth* 124. Elsevier Ltd: 102929. doi:10.1016/j.pce.2020.102929.

Kain, J., J. McConville, and M. Billger. 2021. *Serious games in support of transformative multi-stakeholder sanitation planning for increased resource recovery Specifications for game development*. Gothenburg, Sweden.

Kariuki, R. W., L. K. Munishi, C. J. Courtney-Mustaphi, C. Capitani, A. Shoemaker, P. J. Lane, and R. Marchant. 2021. Integrating stakeholders' perspectives and spatial modelling to develop scenarios of future land use and land cover change in northern Tanzania. *Plos One* 16: 1–31. doi:10.1371/journal.pone.0245516.

Leizeaga, A., C. Duran, L. Hicks, H. Sandén, M. Wondie, and J. Rousk. 2022. Using a tropical elevation gradient to evaluate the impact of land-use intensity and forest restoration on the microbial use of organic matter under climate change. *Global Biogeochemical Cycles* 36: 1–16. doi:10.1029/2021GB007109.

Livsey, J., C. Thi Da, A. Scaini, T. Huynh, T. H. P. Lan, T. X. Long, H. Berg, and S. Manzoni. 2021. Floods, soil and food – Interactions between water management and rice production within An Giang province, Vietnam. *Agriculture, Ecosystems and Environment* 320: 1–12. doi:10.1016/j.agee.2021.107589.

McConville, J. R., E. Kvarnström, A. C. Nordin, H. Jönsson, and C. B. Niwagaba. 2020. Structured Approach for Comparison of Treatment Options for Nutrient-Recovery From Fecal Sludge.

Frontiers in Environmental Science 8: 1–12. doi:10.3389/fenvs.2020.00036.

McConville, J. R., E. Kvarnström, M. Ahlström, and C. B. Niwagaba. 2022. Possibilities for changing to resource recovery in Kampala's on-site sanitation regime. *Resources, Conservation and Recycling* 181. doi:10.1016/j.resconrec.2022.106275.

Mulligan, J., J. Herper, P. Kipkemboi, B. Ngobi, and A. Collins. 2016. Community-responsive adaptation to flooding in Kibera, Kenya. *Engineering Sustainability* 170: 268–280. doi:10.1680/jensu.15.00060.

Mulligan, J., V. Bukachi, R. Gregoriou, N. Venn, D. Ker-Reid, A. Travers, J. Benard, and L. O. Olang. 2019. Participatory flood modelling for negotiation and planning in urban informal settlements. *Engineering Sustainability* 17: 1–18. doi:10.1680/jensu.17.00020.

Rogers, P. M., M. Fridahl, P. Yanda, A. Hansson, N. Pauline, and S. Haikola. 2022. Socio-economic determinants for biochar deployment in the southern highlands of Tanzania. *Energies* 15: 1–19. doi:10.3390/en15010144.

Von Uexkull, N., and H. Buhaug. 2021. Security implications of climate change: A decade of scientific progress. *Journal Of Peace Research* 58: 3–17. doi:10.1177/0022343320984210.

Venkatappa, M., N. Sasaki, and S. Anantsuksomsri. 2020. Applications of the Google Earth Engine and Phenology-Based Threshold Classification Method for Mapping Forest Cover and Carbon Stock. *Remote Sensing* 12: 1–28. doi:10.3390/rs12183110.

Wamsler, C., J. Mulligan, V. Bukachi, and C. Mumbi. 2022. Activating transformation: integrating interior dimensions of climate change in adaptation planning. *Climate and Development*: 1–13. doi:10.1080/17565529.2022.2062287.

Zapata Campos, M. J., S. Carenzo, J.-H. Kain, M. Oloko, J. Pérez Reynosa, and P. Zapata. 2020. Inclusive recycling movements: a green deep democracy from below. *Environment & Urbanization*

7: 1–20. doi:10.1177/0956247820967621.

Zapata Campos, M. J., P. Zapata, and J. Pérez Reynosa. 2023a. (Re)gaining the urban commons: everyday, collective, and identity resistance. *Urban Geography* 44: 1259–1284. doi:10.1080/02723638.2022.2090117.

Zapata Campos, M. J., S. Carenzo, G. Charles, J. Gutberlet, J. H. Kain, M. O. Oloko, J. P. Reynosa, and P. Zapata. 2023b. Grassroots innovations in 'extreme' urban environments. The inclusive recycling movement. *Environment and Planning C: Politics and Space* 41: 351–374. doi:10.1177/23996544221118191.

Zapata Campos, M. J., E. Barinaga, J. H. Kain, M. Oloko, and P. Zapata. 2023c. Organising grassroots infrastructure: The (in)visible work of organisational (in)completeness. *Urban Studies* 60: 126–145. doi:10.1177/00420980211062818.
